# Prediction of cardiovascular mortality based on iron metabolism in patients with cardiovascular–kidney–metabolic syndrome: development and validation of clinical predictive model

**DOI:** 10.1186/s40001-025-03713-x

**Published:** 2026-01-19

**Authors:** Ailifeire Aihaiti, Abulajiang Saidaming, Hasiyeti Tuerxun, Nan Li, Muyesai Nijiati

**Affiliations:** 1 Xinjiang Emergency Center, People’s Hospital of Xinjiang Uygur Autonomous Region, No.91 Tianchi Road, Tianshan District, Urumqi City, Xinjiang Uygur Autonomous Region China; 2 Hospital of Xinjiang Traditional Uygur Medicine, Urumqi, Xinjiang China

**Keywords:** Cardiovascular–kidney–metabolic syndrome, Iron metabolism, Cardiovascular disease mortality, National Health and Nutrition Examination Survey, Prediction model

## Abstract

**Background:**

Iron metabolism is associated with cardiovascular disease (CVD) mortality in patients with cardiovascular–kidney–metabolic (CKM) syndrome. However, its prognostic significance remains unclear. This study was aimed at evaluating incorporation of iron metabolism indicators as a predicting tool for CVD mortality in CKM.

**Methods:**

This study was conducted on data of CKM patients collected from the NHANES between 1999 and 2018. The outcome was CVD mortality. Predictors included iron metabolism indicators and traditional risk factors. Cox proportional hazard models were performed to construct the model. The performance of model was assessed through Harrell’s C, NIR, IDI, AIC, BIC and decision curve analysis curves. The nomogram was depicted to predict CVD mortality risk.

**Results:**

Total of 26,609 patients were included, among which 12,808 (48.1%) were male, and the mean age was 51 years. Over a median follow-up of 9.7 years, there were 911 (3.4%) CVD deaths. Among the iron metabolism indicators, iron and ferritin were associated with CVD mortality, and the performance of model improved after adding to the basic model (Harrell’s C, 0.8353 vs 0.8880; NRI, < 0.001; IDI, < 0.001; AIC, 1667.7860 vs 324.8295; BIC, 1740.5520 vs 390.4293) in the training set. These were confirmed in the validation set. The model also demonstrated improved reclassification and discrimination (Harrell’s C, 0.8332 vs 0.8946; NRI, < 0.001; IDI, 1.333; AIC, 769.3590 vs 195.0582; BIC, 834.3515 vs 250.9120).

**Conclusions:**

The incorporation of serum iron and ferritin levels enhanced the predictive value for CVD mortality in CKM patients without pre-existing CVD.

## Introduction

For decades, cardiovascular disease (CVD) has been the leading cause of death worldwide, accounting for approximately one-third of all mortality [1]. Although the data showed that age-standardized CVD mortality has fallen, the decline has been slowing and nearly stagnant in the past few years [2], and cardiovascular–kidney–metabolic (CKM) syndrome is considered to be a possible contributor [3]. CKM syndrome is a health disorder due to connections among heart diseases, kidney diseases, diabetes and obesity leading to poor health outcomes [4]. Data show that the prevalence of CKM syndrome is high and continues to increase globally [5], which indicates that CVD mortality caused by CKM may also increase. Therefore, early screening of the risk factors and timely intervention are important for improved clinical management of patients with CKM syndrome.

Recent studies have discovered a close relationship between iron metabolism and CVD mortality [6, 7]. Improving iron metabolism can have a good impact on the prognosis of CVD patients [8]. In addition, iron metabolism disorder has also been found to be associated with adverse CVD outcomes in diabetes and chronic kidney diseases [9–11]. Therefore, when evaluating the cardiovascular health status of CKM syndrome patients, it is crucial to consider iron metabolism. However, there is a lack of research that examined the relationship between iron metabolism and the prognosis of CKM syndrome patients. Therefore, the aim of this study is to develop and validate a prediction tool for early identification of the risk factors of CVD in patients with CKM syndrome.

## Methods

### Data source

This cohort study used data from the National Health and Nutrition Examination Survey (NHANES) conducted by the National Center for Health Statistics (NCHS). Participants completed personal interview questionnaires and then received a health examination in the mobile examination center. The NHANES program was approved by the NCHS Research Ethics Review Board, and each respondent signed an informed consent form. NHANES data are released in 2-year cycles. For the present study, we analyzed data from ten released cycles from 1999 to 2018. We used the TRIPOD checklist when writing our report [12].

**Fig. 1:**
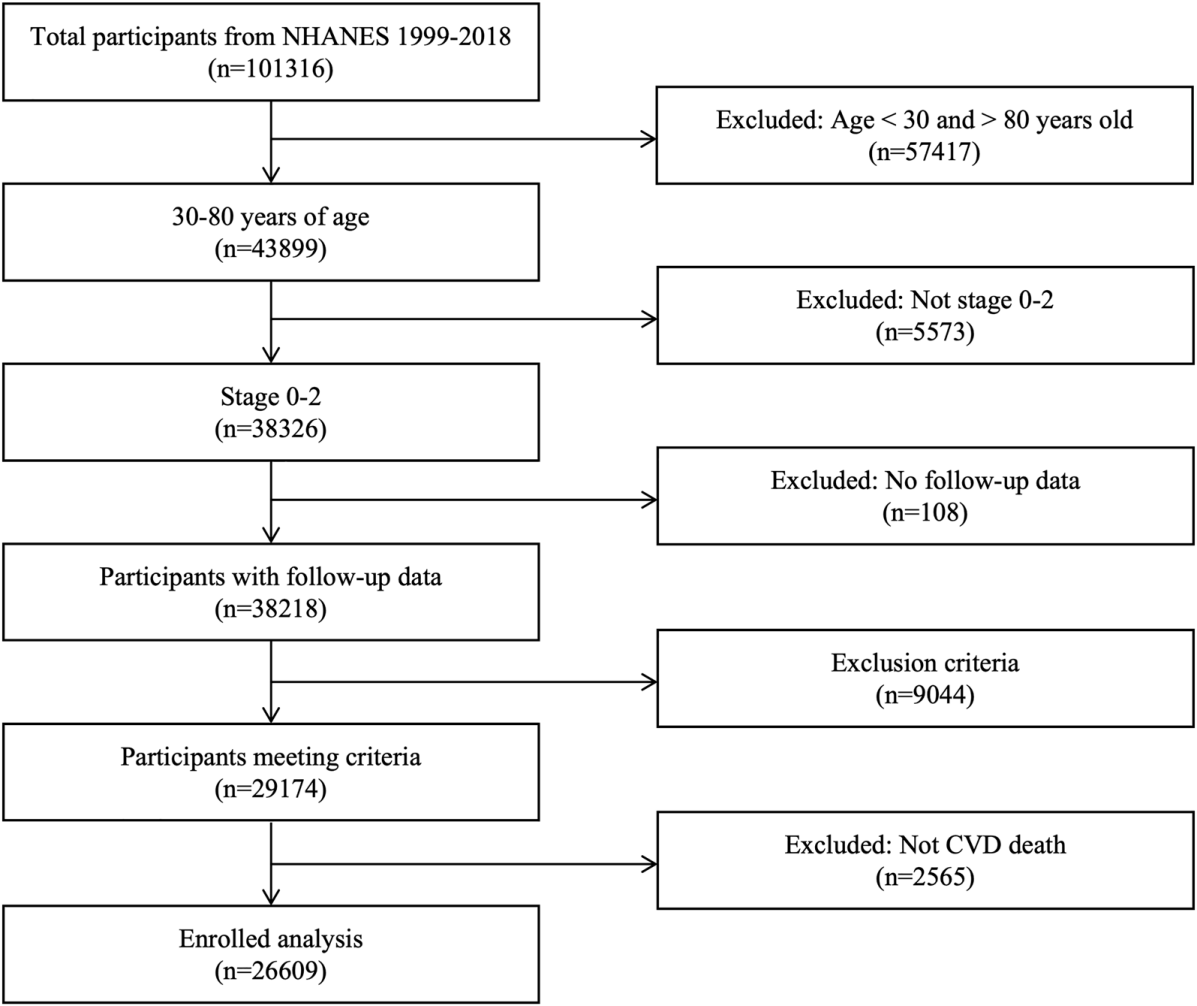
Flowchart of participants included in the analysis

A total of 101,316 individuals completed the NHANES surveys from 1999–2000 to 2017–2018 with 43,899 aged 30–80 years. Of the 43,899 participants in this age group, 26,609 participants syndrome at stage 0–2 CKM without CVD at baseline were included in the analysis, among which, 65% were randomly assigned to the training set and 35% to the validation set. In detail, CKM and corresponding disease stages were defined based on the recommendations in the Presidential Advisory from the American Heart Association [4]. In addition, by referring to this advisory, we excluded people whose systolic blood pressure (SBP) was less than 90 or greater than 200 mmHg, total cholesterol level less than 130 or greater than 320 mg/dl, body mass index (BMI) less than 18.5 or greater than 40 kg/m^2^. The flow diagram of the study inclusion and exclusion process is presented in Fig. 1.

### Assessment of CVD mortality

The analysis outcome was CVD-specific mortality, which were derived from the National Death Index. The follow-up period was calculated from the date of examination until death or the end of the follow-up, whichever occurred first. Death from CVD was identified as codes I00–09, I11, I13, I20–51, and I60–69 using the International Classification of Diseases, Tenth Revision (ICD-10).

### Predictors of CVD mortality

The variables included were as follows: sex, age, race, smoking, diabetes, anti-hypertensive use, statin use, BMI, SBP, estimated glomerular filtration rate (eGFR), total cholesterol (TC), high density lipoprotein cholesterol (HDL), low density lipoprotein cholesterol (LDL), serum iron, total iron binding capacity (TIBC), ferritin and transferrin saturation (TS).

Variables such as sex, age, race, smoking, diabetes, anti-hypertensive use and statin use were adopted from demographic and health questionnaires. BMI and SBP were obtained from health examination. eGFR, TC, HDL, LDL, iron, TIBC, ferritin and TS were acquired from laboratory results.

### Variables definition

Smoking status was categorized as never (smoked less than 100 cigarettes in life), ever (smoked more than 100 cigarettes but not smoking at all now) and current (smoked more than 100 cigarettes and smoking some days or every day now). Diabetes was defined as a self-reported history of diabetes, use of diabetes medication or insulin, glycosylated hemoglobin (HbA1c) ≥ 6.5%, fasting plasma glucose ≥ 7 mmol/L and random blood glucose ≥ 11.1 mmol/L. Anti-hypertensive use was self-reported. Use of statins was determined through a pill bottle review that was conducted as part of the study examination. During the household interview, survey participants were asked to show all prescription medications taken in the past 30 days. Statins included atorvastatin, simvastatin, rosuvastatin, fluvastatin, pitavastatin, lovastatin, and cerivastatin. TS was calculated as iron divided by TIBC. The eGFR was calculated using the chronic kidney disease–epidemiology collaboration (CKD–EPI) equation.

### Statistical analysis

Continuous variables are presented as mean ± standard deviation or median (interquartile range), based on their distribution normality and compared between the two sets using Student’s *t* test or Mann–Whitney test, respectively. Categorical variables were expressed as number (percentage) and compared using *χ2* test. The basic model to predict risk of CVD mortality included the following predictors refer to the PREVENT model [13]: sex, age, smoking, diabetes, anti-hypertensive use, statin use, BMI, SBP, eGFR, TC, HDL and LDL. The iron metabolism variables in the training set underwent a filtering process through univariate and multivariable cox regression analysis.

The accuracy of the prediction model was assessed from the discrimination and calibration sides through several metrics, including Harrell’s C, net reclassification index (NIR), integrated discrimination improvement (IDI), akaike information criterion (AIC), Bayesian information criteria (BIC) and decision curve analysis (DCA) curves. These evaluations were conducted separately for the training and validation sets. Finally, a nomogram was depicted to predict CVD-specific mortality risk based on significant attributes among CKM patients in stages 0–2.

All *p* values are two-sided and considered significant when *P* < 0.05. All data arrangements and analysis were performed using Stata 17.0 and R studio software (4.3.1).

## Results

### Baseline characteristics of the study population categorized by training and validation sets

Among all participants, the mean age was 51 years, comprising 12,808 (48.1%) men and 13,801 (51.9%) women. Over a median follow-up period of 9.7 years, 911 (3.4%) CVD deaths occurred. Within the training set, the mean age of participants was 51 years, consisting of 8,389 (48.5%) men and 8,907 (51.5%) women. Over a median follow-up period of 9.6 years, 602 (3.5%) CVD deaths occurred. Within the validation set, the mean age was 51 years, consisting of 4,419(47.5%) men and 4,894 (52.6%) women. Over a median follow-up period of 9.8 years, 309 (3.3%) CVD deaths occurred. When comparing the two groups, there were no statistically significant variations, as shown in Table 1.

**Table 1:** Baseline characteristics of training and validation sets^a,b^

Characteristics	Training set	Validation set	*p* value
Sex			
Female	8907 (51.5%)	4894 (52.6%)	0.101
Male	8389 (48.5%)	4419 (47.4%)	
Age (years)	50 (39, 62)	50 (39, 62)	0.464
Race			
Mexican American	3211 (18.6%)	1721 (18.5%)	0.449
Other Hispanic	1632 (9.4%)	845 (9.1%)	
Non-Hispanic white	7470 (43.2%)	4025 (43.2%)	
Non-Hispanic black	3272 (18.9%)	1739 (18.7%)	
Other races	1711 (9.9%)	983 (10.6%)	
Smoking			
Never	9571 (55.4%)	5260 (56.5%)	0.116
Ever	4316 (25.0%)	2223 (23.9%)	
Current	3392 (19.6%)	1826 (19.6%)	
Diabetes (%)			
No	7073 (74.3%)	3822 (74.4%)	0.937
Yes	2443 (25.7%)	1316 (25.6%)	
Anti-hypertensive use			
No	4038 (86.7%)	2153 (84.9%)	0.034
Yes	619 (13.3%)	383 (15.1%)	
Statin use			
No	7060 (74.3%)	3777 (73.9%)	0.591
Yes	2441 (25.7%)	1334 (26.1%)	
BMI (kg/m^2^)	27.9 (24.7, 31.6)	27.8 (24.6, 31.5)	0.335
SBP (mmHg)	122 (112, 134)	122(112, 134)	0.559
eGFR (ml/min)	107.3 (88.8, 132.9)	107.7 (89.0, 133.2)	0.460
TC (mg/dl)	199.0 (175.0, 226.0)	200.0 (176.0, 227.0)	0.302
HDL (mg/dl)	51.0 (42.0, 63.0)	51.0 (42.0, 63.0)	0.677
LDL (mg/dl)	118.0 (97.0, 141.0)	120.0 (98.0, 143.0)	0.056
Iron (umol/L)	14.9 (11.3, 19.0)	14.7 (11.3, 18.8)	0.475
TIBC (umol/L)	61.8 (55.5, 69.5)	61.9 (55.5, 68.7)	0.415
Ferritin (ug/L)	73.0 (32.0, 153.0)	72.6 (32.0, 149.0)	0.457
TS	24.4 (18.0, 32.1)	24.1 (17.9, 31.4)	0.217

*BMI* body mass index, *SBP* systolic blood pressure, *eGFR* estimated glomerular filtration rate, *TC* total cholesterol, *HDL* high density lipoprotein cholesterol, *LDL* low density lipoprotein cholesterol, *TIBC* total iron binding capacity, *TS* transferrin saturation

^a^Data are expressed as median (inter-quartile range) or number (percentage) where appropriate

^b^*P was calculated by the Mann–Whitney test for continuous variables and the chi-squared test for categorical variables

### Construction of clinical prediction models

To develop clinical prediction models, we employed cox regression to analyze data from the training set. First, we used univariate regression analysis, and found that iron, TIBC and ferritin were the significant influencing factors (all *P* < 0.05). After performing multivariate cox regression analysis, only iron and ferritin (all *P* < 0.05) were selected (Table 2).

**Table 2:** Risk prediction for CVD specific mortality

Risk factors	HR, 95% CI *P*	Adjusted HR, 95% CI *P*
Iron (umol/L)	0.976, 0.963–0.989, < 0.001	0.937, 0.904–0.972, < 0.001
TIBC (umol/L)	0.979, 0.967–0.992, 0.001	0.973, 0.939–1.008, 0.127
Ferritin (ug/L)	1.002, 1.001–1.003, < 0.001	1.005, 1.002–1.007, 0.002
TS	0.999, 0.988–1.011, 0.925	0.992, 0.958–1.027, 0.632

*TIBC* total Iron binding capacity, *TS* transferrin saturation

### Validation of clinical prediction models

**Fig. 2:**
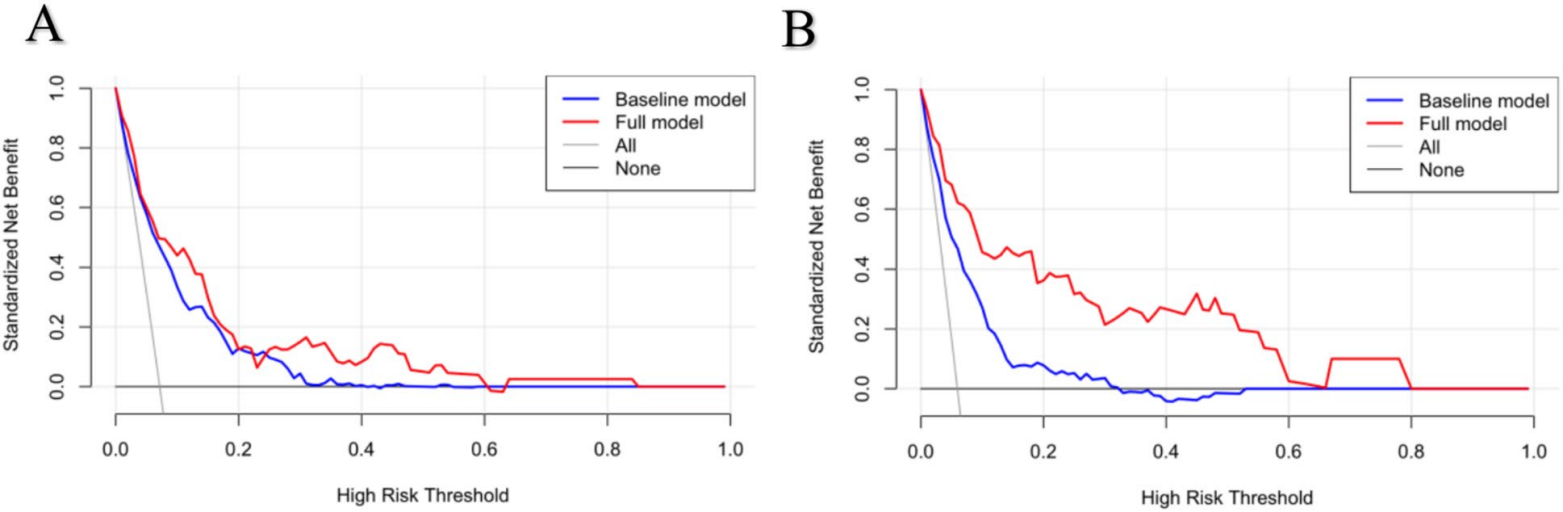
Decision curves for the basic model and the full model in both derivation (**A**) and validation (**B**) groups

To assess the reliability of the established prediction model, we used a series of statistical indicators. For discrimination (Table 3), compared with the basic model, the Harrell’s C of full model were higher in the training cohort (0.8756 vs 0.8188) and the validation cohort (0.8913 vs 0.8261), which indicated that the prediction ability was in good accordance with the real observation. NRI and IDI of full model revealed significant improvement in both cohorts (NRI, 0.285, 0.034; IDI, 0.047, 0.026). For calibration (Table 3), compared with the basic model, full model has lower AIC and BIC values. The decision curve analysis (DCA) showed better benefits for the prediction of CVD mortality whether in the training set (Fig. 2A) or the validation set (Fig. 2B) within a wide threshold probability range.

**Table 3:** Predictive accuracy of baseline risk model and full model derivatives for CVD-specific mortality in both derivation and validation groups

Statistics		Training set		Validation set	
		Basic model^a^	Full model^b^	Basic model	Full model
Discrimination	Harrell’s C	0.8353	0.8880	0.8332	0.8946
	NRI (*p* value)	Reference	< 0.001	Reference	< 0.001
	IDI (*p* value)	Reference	< 0.001	Reference	1.333
Calibration	AIC	1667.7860	324.8295	769.3590	195.0582
	BIC	1740.5520	390.4293	834.3515	250.9120

*AIC* akaike information criterion, *BIC* Bayesian information criteria, *NIR* net reclassification index, *IDI* integrated discrimination improvement

^a^The basic model included sex, age, , smoking, diabetes, anti-hypertensive use, statin use, BMI, SBP, eGFR, TC, HDL and LDL

^b^The full model adjusted based on the basic model and added variables, including iron and ferritin

### Nomogram

**Fig. 3:**
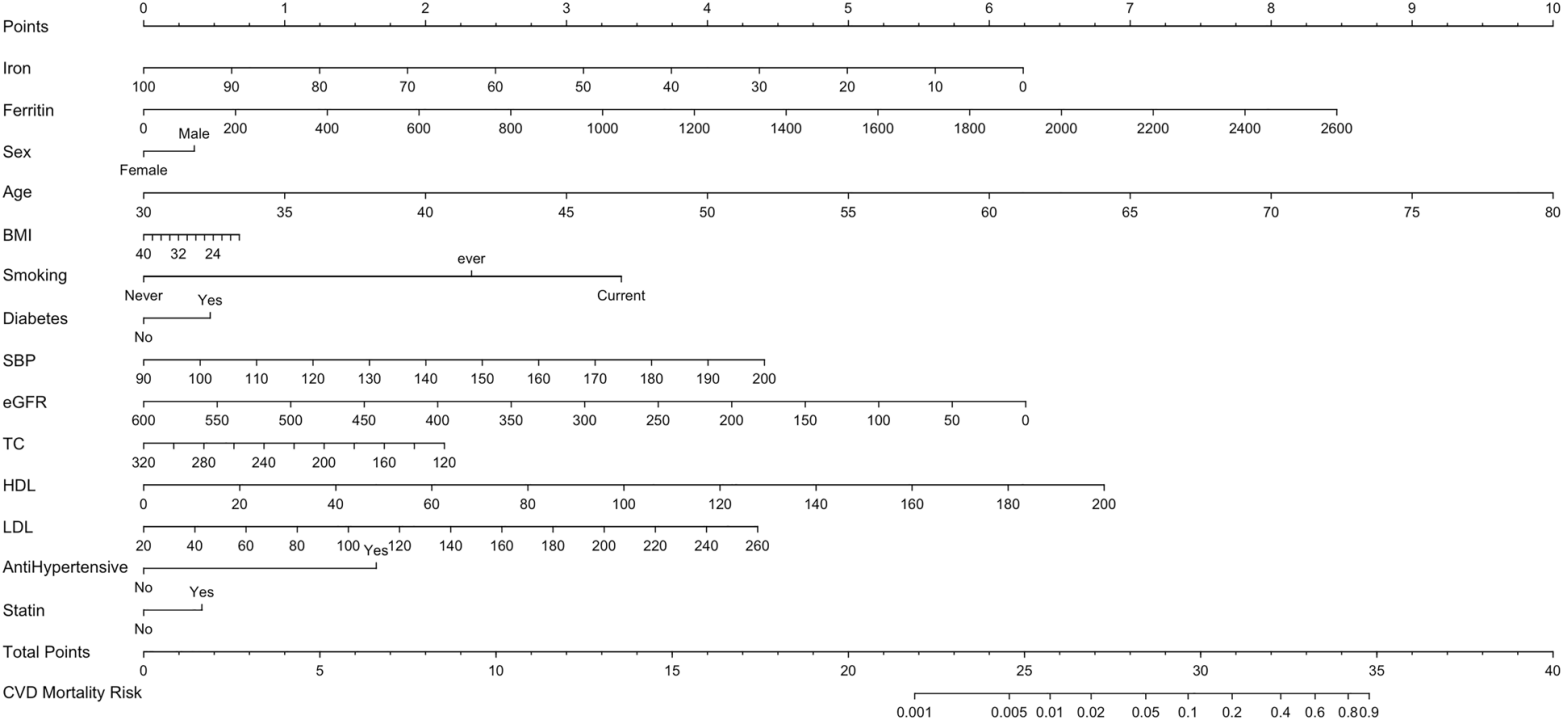
Nomogram

Finally, the nomogram model was established using predictive variables filtered by multivariate cox regression (Fig. 3). Corresponding to each risk factor, a single score could be obtained. Every single score was summed, and the total score corresponded downward to the probability of CVD mortality in this patient.

### Risk calculation formula

The risk score can be calculated using the following formula:

Linear Predictor (LP) = −10.770754−0.037445129 × Iron + 0.0019537858 × Ferritin + 0.2152142 × (Sex = "Male") + 0.12000592 × Age−0.018496541 × BMI + 1.3959375 × (Smoking = "ever") + 2.0334828 × (Smoking = "Current") + 0.28311307 × (Diabetes = "Yes") + 0.024025624 × SBP−0.0062588378 * eGFR−0.0064011044 × TC + 0.020446687 × HDL + 0.010892174 × LDL + 0.98980381 × (Anti-hypertensive use= "Yes") + 0.24751954 × (Statin use= "Yes"), Risk = 1−S_0_(t)^exp(LP),

where S_0_(t) = 0.993 represents the baseline survival probability at 9.6 years, corresponding to the median follow-up time in our training cohort.

## Discussion

Iron metabolism disturbances are commonly seen among CKM syndrome patients, and associated with poor CVD outcomes, but are often overlooked in clinical practice. This study was conducted to develop and validate a model to predict CVD mortality for 30–80-year-old CKM syndrome adults without CVD at baseline. Among the iron metabolism indicators, serum iron and ferritin were found to be independent predictors and used to construct our prognostic model by combining the traditional risk factors. The statistical analysis showed that the model demonstrated improved discrimination and calibration after adding the serum iron and ferritin.

In this study, serum iron was negatively associated with the CVD mortality of CKM syndrome patients. Although there are few studies on serum iron and cardiovascular prognosis of patients with CKM syndrome, the correlation between serum iron and cardiovascular risk factors including metabolic risks was demonstrated. Gutierrez-Bedmar et al. reported that serum iron has inverse associations with CVD events for population at high cardiovascular risks [7]. Among individuals with coronary artery disease, increased serum iron was found to have a protective role [14], and in patients with diabetes, it was associated with a decreased risk of CVD mortality [15], both consistent with our findings in the CKM population. As for incident of metabolic syndrome, a negative association with iron was found in women [16]. In a nationally representative survey in China, impaired glucose metabolism group had lower serum iron level than the normal group [17]. The protective role of serum iron may stem from its representation of the circulating, immediately available iron pool that is crucial for fundamental processes, such as erythropoiesis and cellular respiration [18, 19]. Adequate circulating iron ensures optimal oxygen delivery and energy metabolism in cardiomyocytes and the vascular system, thereby protecting against CVD.

Serum ferritin is another predictor in this study, and the higher its level, the higher the risk of death. At present, a large number of research has proved that ferritin is positively correlated with mortality in patients with chronic diseases, such as heart diseases and kidney diseases [20, 21]. In recent years, studies also found the same relationship in the general population. In the English longitudinal study of aging, high ferritin levels in men with no major chronic diseases were independently associated with an increased risk of CVD mortality [22]. In a Danish population study, a significantly higher rate of CVD mortality in participants with increased ferritin levels was observed [23]. A Recent meta-analysis which included 14 studies and 74,710 samples reported a higher risk of metabolic syndrome in patients with increased serum ferritin [24]. A Chinese population-based study pointed out that in patients at risk of obesity, high ferritin levels exhibited worse CVD risk profiles [25]. The underlying mechanism is likely twofold. First, ferritin is the primary iron storage protein, and elevated levels indicate increased body iron stores, which can promote oxidative stress via the Fenton reaction [26]. Second, and perhaps more critically, ferritin is an acute-phase reactant, and its elevation is a marker of underlying inflammation, a well-established driver of atherosclerosis and CVD progression [27, 28]. Moreover, ferritin’s close relationship with lipids [29], which may be related to inflammation and lipid peroxidation caused by iron metabolism disorders [30], provides a plausible pathophysiological link that our model captures.

Many iron status indicators have been used to assess the relation of iron metabolism to CVD risk. A key and nuanced finding of our analysis is that although both serum iron and ferritin emerged as independent predictors, TS was not retained in the final model. This does not diminish the clinical value of TS in diagnosing functional iron deficiency, but rather suggests that for long-term mortality risk stratification in CKM syndrome. The model derives more prognostic power from evaluating circulating iron and storage or inflammatory iron separately, rather than combining them into the ratio that is transferrin saturation. We hypothesize that this is because serum iron and ferritin capture distinct, non-redundant pathophysiological pathways. In the complex inflammatory milieu of CKM syndrome, transferrin saturation can be confounded: a patient with concurrent inflammation, which elevates ferritin, and functional iron deficiency, which lowers serum iron, might present with a deceptively normal transferrin saturation, thereby masking two significant, competing risk pathways. Thus, our model underscores that in CKM, both functional iron availability, best captured directly by serum iron, and the iron storage or inflammatory burden, captured by ferritin, are critical, and their independent contributions are best captured by including them separately, rather than relying solely on transferrin saturation.

In addition, our basic model was consistent with PREVENT base model published by the American Heart Association which supports the potential clinical relevance of our modeling approach. The integration of iron and ferritin into this established framework suggests a potential, readily accessible approach to refine risk stratification in CKM patients.

### Limitation

There are some limitations in this study. First, although the data were derived from a large-scale national survey (NHANES), they originate from a single country, which may limit generalizability. Second, our prediction model has not undergone external validation, which we will address in future research. Third, we selected only four iron status indicators among numerous possible indicators that can provide insights into iron metabolism status based on the fact that they are easily accessible in clinical settings.

## Conclusion

Epidemiological data showed that iron metabolism is common in patients with CKM syndrome, but few prognostic studies showed promising factors. Our prediction model, which incorporates serum iron and ferritin, demonstrated good predictive accuracy for CVD death in this study cohort of CKM syndrome patients. Further clinical validations will be needed in future studies.

## Data Availability

The datasets analyzed during the current study are available in the NHANES dataset [https://wwwn.cdc.gov/nchs/nhanes/Default.aspx].

## Abbreviations

CKM: Cardiovascular–kidney–metabolic syndrome
NHANES: National Health and Nutrition Examination Survey
CVD: Cardiovascular disease
NIR: Net reclassification index
IDI: Integrated discrimination improvement
AIC: Akaike information criterion
BIC: Bayesian information criteria
DCA: Decision curve analysis
NCHS: National Center for Health Statistics
BMI: Body mass index
ICD-10: International Classification of Diseases, Tenth Revision
SBP: Systolic blood pressure
eGFR: Estimated glomerular filtration rate
TC: Total cholesterol
HDL: High density lipoprotein cholesterol
LDL: Low density lipoprotein cholesterol
TIBC: Total iron binding capacity
TS: Transferrin saturation
CKD–EPI: The chronic kidney disease–epidemiology collaboration
